# Effects of Cold Exposure on Performance and Skeletal Muscle Fiber in Weaned Piglets

**DOI:** 10.3390/ani11072148

**Published:** 2021-07-20

**Authors:** Jie Yu, Shuai Chen, Ziyou Zeng, Shuaibing Xing, Daiwen Chen, Bing Yu, Jun He, Zhiqing Huang, Yuheng Luo, Ping Zheng, Xiangbing Mao, Junqiu Luo, Hui Yan

**Affiliations:** 1 Key Laboratory for Animal Disease-Resistance Nutrition of Sichuan Province, Animal Nutrition Institute, Sichuan Agricultural University, Chengdu 611130, China; chenshuai@tianzow.com (S.C.); xsb19951120@163.com (S.X.); dwchen@sicau.edu.cn (D.C.); ybingtian@163.com (B.Y.); hejun8067@163.com (J.H.); zqhuang@sicau.edu.cn (Z.H.); luoluo212@126.com (Y.L.); zpind05@163.com (P.Z.); acatmxb2003@163.com (X.M.); 13910@sicau.edu.cn (J.L.); yan.hui@sicau.edu.cn (H.Y.); 2 Sichuan Tequ Agriculture and Animal Husbandry Technology Group Co., Ltd., Chengdu 610207, China; ziyouzeng@foxmail.com

**Keywords:** weaned piglets, cold exposure, growth performance, skeletal muscle fiber, antioxidant capacity

## Abstract

**Simple Summary:**

Muscle fiber is the basic unit of muscle composition. The type of skeletal muscle fiber can be transformed from fast-twitch to slow-switch or vice versa by internal and external factors. Low-temperature is one of the major environmental factors that influences the growth performance of animals. However, the influence of low-temperature on weaned piglets’ skeletal muscle fiber, and whether this influence is related to mitochondrial function and antioxidant capacity, has not been reported. Our results indicated that low temperature could negatively affect growth performance and nutrient digestibility in weaned piglets. Moreover, evidence was provided to show that low-temperature induces a shift toward oxidative muscle fibers, which may occur through mitochondrial function regulation and increased antioxidative capacity.

**Abstract:**

Low-temperature is one of the most significant risks for the animal industry. In light of this, the present study aimed to explore the effects of low-temperature on growth performance, nutrient digestibility, myofiber types and mitochondrial function in weaned piglets. A total of sixteen 21-day-old male Duroc × Landrace × Yorkshire (DLY) piglets were randomly divided into a control group (CON, 26 ± 1 °C) and a low-temperature group (LT, 15 ± 1 °C), with eight duplicate piglets in each group. The trial period lasted for 21 days. We showed that LT not only increased the ADFI (*p* < 0.05), as well as increasing the diarrhea incidence and diarrhea index of weaned piglets in the early stage of the experiment (*p* < 0.01), but it also decreased the apparent digestibility of crude protein (CP), organic matter (OM) and dry matter (DM) (*p* < 0.05). Meanwhile, in the LT group, the mRNA expression of *MyHC IIa* (*p* < 0.05) in longissimus dorsi muscle (LM) and *MyHC I* (*p* < 0.01) in psoas muscle (PM) were increased, while the mRNA expression of *MyHC IIx* in PM was decreased (*p* < 0.05). In addition, LT increased the mRNA expression of mitochondrial function-related genes citrate synthase (CS) and succinate dehydrogenase-b (SDHB) in LM, as well as increased the mRNA expression of CS (*p* < 0.05) and carnitine palmitoyl transferase-1b (CPT-1b) (*p* < 0.01) in PM. Furthermore, LT increased the T-AOC activity in serum and LM (*p* < 0.01), as well as increased the T-SOD activity in PM (*p* < 0.05). Taken together, these findings showed that low-temperature could negatively affect the growth performance and nutrient digestibility, but resulted in a shift toward oxidative muscle fibers, which may occur through mitochondrial function regulation.

## 1. Introduction

Temperature is one of the main environmental factors that influences the growth and development of animals. Low ambient temperature not only affects growth performance, but also reduces antioxidant capacity and immunity of young animals [1,2]. In cold environments, animals mainly rely on two ways to produce heat to maintain body temperature, largely shivering thermogenesis and non-shivering thermogenesis; shivering thermogenesis means to produce heat by the contraction of skeletal muscle, and non-shivering thermogenesis means to produce heat by nutrient metabolism [3]. Skeletal muscle is composed of a large number of muscle fibers. Different types of skeletal muscle fibers have different energy metabolisms and contraction speeds [4]. The mammalian skeletal muscle can be divided into four fiber types including type I with *MyHC I*, type II with *MyHC IIa*, *MyHC IIx* and *MyHC IIb* [5]. Previous research found that type I fibers have lower excitation thresholds and stronger oxidative metabolism capacity than type II fibers [6]. Although shivering thermogenesis of skeletal muscle is indispensable for piglets to maintain body temperature in the LT environment, there is little known about the effects of cold exposure on skeletal muscle characteristics. Therefore, in the present study, our aim was to investigate the effects of LT on growth performance, antioxidant capacity, myofiber types and mitochondrial function in weaned piglets.

## 2. Materials and Methods

### 2.1. Animals, Diets and Experimental Design

Sixteen 21-day-old male Duroc × Landrace × Yorkshire (DLY) piglets with an average body weight of 6.5 ± 0.5 kg were randomly divided into 2 groups with 8 duplicates. The animals were housed individually in pens under low temperature (LT, 15 ± 1 °C) or thermoneutral temperature (CON, 26 ± 1 °C). The thermoneutral temperature (CON, 26 ± 1 °C) in the experiment was according to the recommendations of Dividich et al. [7]. The diet was formulated according to NRC (2012). Ingredients and calculated nutrient contents of the diet were presented in Table 1. The trial period lasted for 21 days. Food and water were provided ad libitum throughout the experiment.

### 2.2. Growth Performance

Feed intake was recorded daily. The pigs were individually weighed at the start and the end of the trial to calculate average daily body weight gain (ADG), average daily feed intake (ADFI) and the ratio of feed to gain (F/G).

### 2.3. Diarrhea Score

The diarrhea score for each pig was monitored between 12 noon and 1 pm daily. The normal consistency of feces formed (no diarrhea) score is 0; the soft, partially formed feces (mild diarrhea) score is 1; the loose, semi-liquid feces (moderate diarrhea) score is 2; and the watery feces (severe diarrhea) score is 3. The average diarrhea incidence and diarrhea score per group was calculated daily.

### 2.4. Sample Collection

At the start of trial, experimental diets were sampled for nutrient digestibility analysis. In the last 4 days of trial, fresh fecal samples from total of 16 pigs were collected immediately after defecation and then placed in individual plastic bags. After collection, 10 mL of a 10% H_2_SO_4_ solution was added to each 100 g of wet fecal sample. All diet and fecal samples were immediately stored at −20 °C for further analysis. At the end of trial, after fasting for 12 h, 10 mL of anterior vena cava blood was taken from each piglet (an empty stomach in the morning), and placed in an inclined position at room temperature for 60 min. The blood sample was collected by centrifugation with 3000× *g* for 10 min at 4 °C prior to antioxidant status analysis. After blood sampling, control group and low temperature group piglets were killed in rotation (first, one pig from the control group was killed, and then one pig from the low temperature group was killed, and then repeated in this order). All piglets were given sodium pentobarbital (200 mg kg^−1^ BW), exsanguinated, dehaired, eviscerated, and split down the muscle. The samples of LM and PM were collected immediately, frozen in liquid nitrogen and then stored at −80 °C until analysis.

### 2.5. Chemical Analysis

The apparent nutrient digestibility was measured, using acid insoluble ash (AIA) as the indicator. For AIA determination, 5 g of finely ground feed or feces was boiled in 75 mL HCl for 15 min, then filtered through ashless filter paper, washed with boiling water until free of acid and finally ashed at 550 °C in a muffle furnace for 8 h [8]. After AIA determination, all diet and fecal samples were analyzed for crude protein, crude ash and dry matter [9]. Gross energy was measured by an automatic adiabatic oxygen bomb calorimeter (Parr Instrument Co., Moline, IL, USA). The apparent nutrient digestibility was calculated uing following formula, where A1 represents the AIA content of the diet; A2 represents the AIA content of feces; F1 represents the nutrient content of the diet and F2 represents the nutrient content of feces:

$$Apparent\;nutrient\;digestibility\;\left(\%\right)=\left(1-\frac{A1*F2}{A2*F1}\right)*100$$

### 2.6. Determination of Antioxidant Parameters in Serum and Skeletal Muscle

About 0.8 g of skeletal muscle sample (LM and PM) was quickly weighed, thawed, and homogenized (1:9, wt/vol) with ice-cold physiological saline using a homogenizer. After this, the mixture of muscle and normal saline was centrifuged at 4000× *g* for 15 min at 4 °C. The supernatant was acquired and stored at −80 °C and used for antioxidant-related enzyme activity examination. Total protein concentration was determined by the BCA Protein Assay Kit (Pierce, Rockford, IL, USA). The total antioxidant capacity (T-AOC), total superoxide dismutase (T-SOD) and malondialdehyde (MDA) level in serum and muscle were determined by commercial kits (Nanjing Jiancheng Bioengineering Institute, Nanjing, China) according to the manufacturer’s instructions.

### 2.7. Total RNA Extraction, Reverse Transcription and Quantitative Real-Time PCR

Total RNA was extracted from samples of the skeletal muscle using the TRIzol reagent (TaKaRa), according to the manufacturer’s instructions. The concentration of RNA in the final preparations was calculated from the OD260. Reverse transcription was performed using the PrimeScript TM RT Reagent Kit (TaKaRa) with a 1 μg RNA sample, according to the manufacturer’s instructions. Complementary DNA was used as the template for PCR. Real-time quantitative PCR was performed in an Option Monitor 3 Real-Time PCR Detection System (Bio-Rad) using the SYBR Green Supermix (TaKaRa). The gene-specific primers used are listed in Table 2. The thermal cycling conditions were 40 cycles of 95 °C for 5 s, and 60 °C for 30 s.

For normalization, β-actin was chosen as the reference gene because no variation in its expression was observed between treatments. The relative mRNA abundance of the analyzed genes was calculated using the 2^−^^△△Ct^ method [10], and the messenger RNA (mRNA) level of each target gene for the CON group was set to 1.0.

### 2.8. Statistical Analysis

Data were analyzed by *t*-test using the statistical program SAS (version 9.4; SAS Inst. Inc., Cary, NC, USA). Each pig served as a statistical unit. Data are presented as the mean ± standard error, and results are reported as least square means and considered extremely significant if *p* ≤ 0.01, significant if *p* ≤ 0.05 and a tendency if 0.05 < *p* ≤ 0.10.

## 3. Results

### 3.1. Growth Performance

Compared with the CON group, LT had no effect (*p* > 0.10) on ADG, but resulted in greater (0.05< *p* ≤ 0.10) F/G, and increased (*p* < 0.05) ADFI (Table 3).

### 3.2. Diarrhea Score

As shown in Table 4, compared with the CON group, diarrhea incidence and diarrhea index tended to increase (0.05 < *p* ≤ 0.10) in the LT group. Moreover, there were significant increases (*p* < 0.01) in diarrhea incidence and diarrhea index during the first week, but there was no significant effect (*p* > 0.05) during the second and third week.

### 3.3. Nutrient Digestibility

As shown in Table 5, compared with the CON group, the apparent digestibilities of CP, OM and DM were decreased (*p* < 0.05) in the LT group. In addition, compared with the CON group, LT has a tendency to decrease the apparent digestibility of GE (0.05 < *p* ≤ 0.10).

### 3.4. Skeletal Muscle Fiber Type-Related Gene Expression

**Figure 1:**
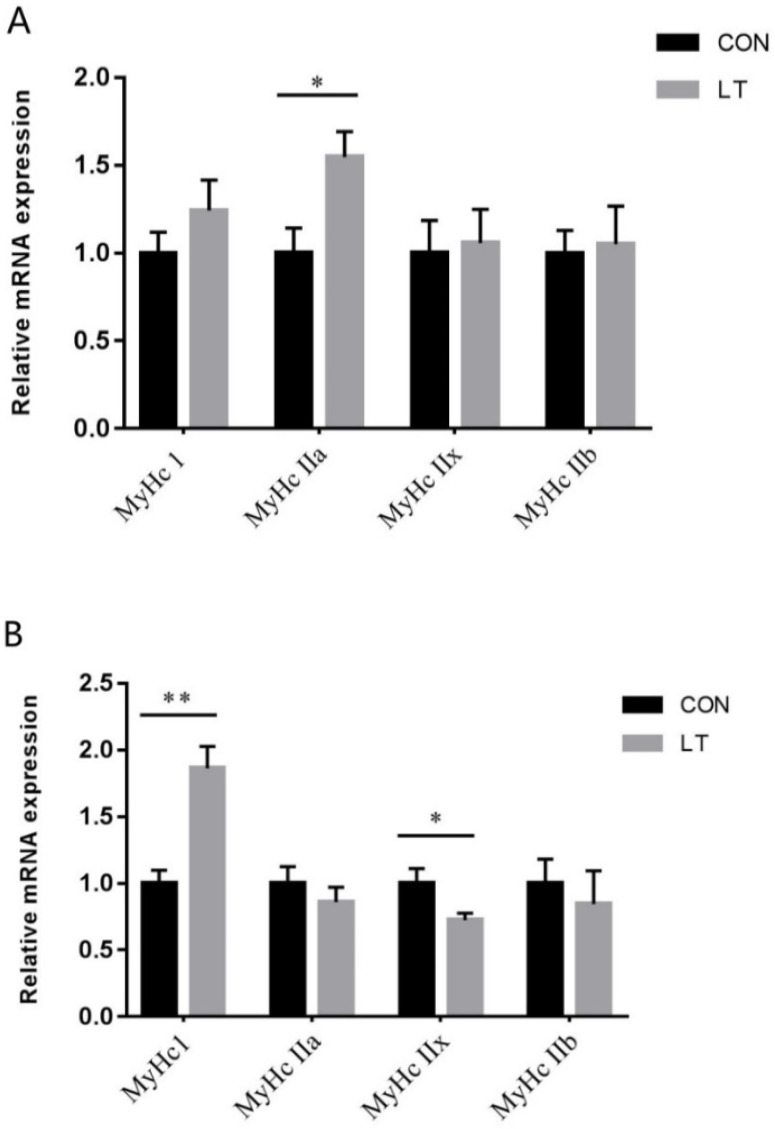
Effects of low-temperature on the mRNA expression level of fiber type-related genes in (**A**) LM and (**B**) PM of weaned piglets. Results were the mean and standard errors. LM, longissimus dorsi muscle; PM, psoas muscle. * Mean values were significantly different between two groups (*p* < 0.05). ** Mean values were very significantly different between two groups (*p* < 0.01).

The *MyHC I*, *MyHC IIa*, *MyHC IIb* and *MyHC IIx* mRNA expressions were detected by real-time quantitative PCR. As shown in Figure 1A, LT had no effect (*p* > 0.05) on LM *MyHC I*, *MyHC IIb* and *MyHC IIx* mRNA levels, but resulted in greater (*p* < 0.05) *MyHC IIa* mRNA levels. As shown in Figure 1B, LT had no effect (*p* > 0.05) on PM *MyHC IIa* and *MyHC IIb* mRNA levels, but resulted in greater (*p* < 0.01) *MyHC I* mRNA levels and lower (*p* < 0.05) *MyHCIIx* mRNA levels.

### 3.5. Mitochondrial Function-Related Gene Expression

**Figure 2:**
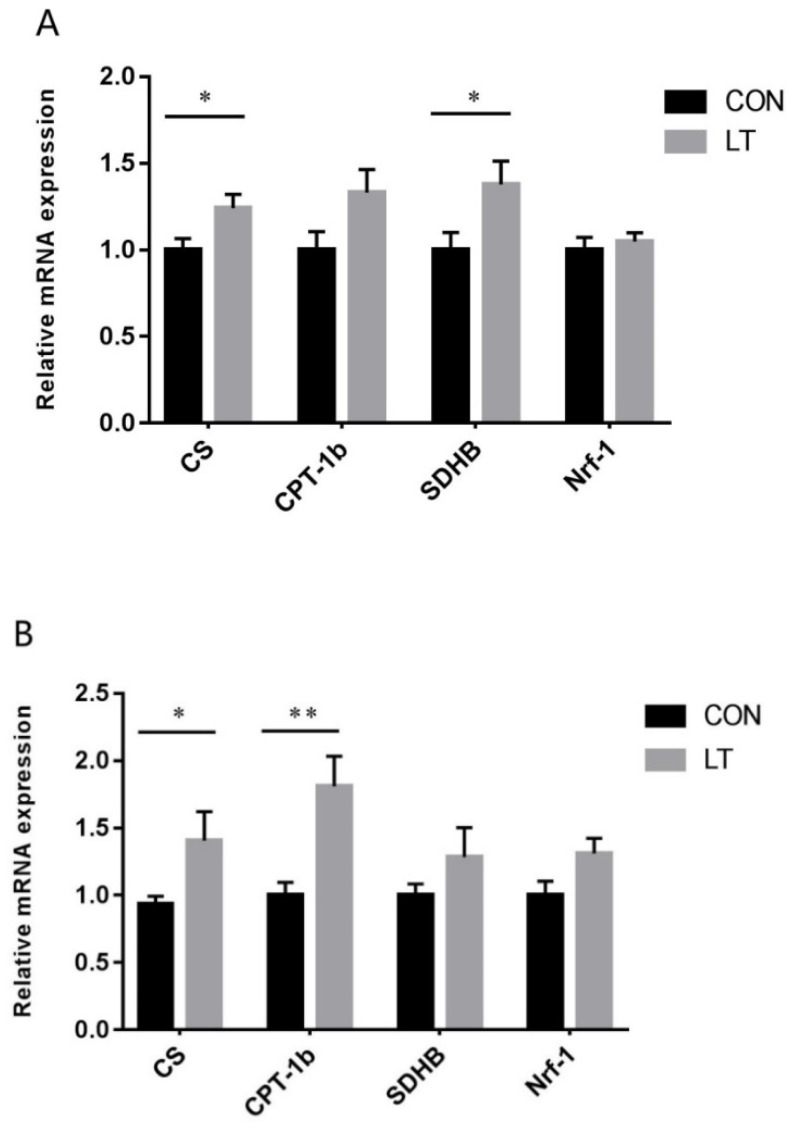
Effects of low-temperature on the mRNA expression level of mitochondrial function-related genes in (**A**) LM and (**B**) PM of weaned piglets. Results were the mean and standard errors. CS, citrate synthase; CPT-1b, carnitine palmitoyl transferase-1b; SDHB, succinate dehydrogenase-b; Nrf-1, nuclear respiratory factor 1. * Mean values were significantly different between two groups (*p* < 0.05). ** Mean values were very significantly different between two groups (*p* < 0.01).

As shown in Figure 2A, compared with the CON group, LT increased (*p* < 0.05) the mRNA expression of CS and SDHB in LM, but had no effect (*p* > 0.05) on CPT-1b and Nrf-1 mRNA levels. As shown in Figure 2B, increased CS (*p* < 0.05) and CPT-1b (*p* < 0.01) mRNA levels were observed in the LT group in PM, but there were no effects (*p* > 0.05) on SDHB and Nrf-1 mRNA levels.

### 3.6. Antioxidant Capacity

The data showed that LT had significantly increased (*p* < 0.01) T-AOC activity in serum (Table 6), while there was no difference (*p* > 0.05) in T-SOD activity and MDA content in serum between the LT group and the control group (Table 6). In LM, LT had no effect (*p* > 0.05) on MDA content and T-SOD activity, but resulted in greater (*p* < 0.01) T-AOC activity (Table 6). The result showed that LT significantly increased (*p* < 0.05) the T-SOD activity in PM, while there was no difference in (*p* > 0.05) T-AOC activity and MDA content in PM between the LT group and the control group (Table 6).

## 4. Discussion

Previous studies have shown that livestock and poultry need to increase heat production by increasing feed intake and body energy metabolism in order to maintain body temperature in the LT environment, which may result in reduced performance and high cost. The present study showed that the F/G of weaned piglets increased in the LT environment, which was consistent with the results obtained in growing-finishing pigs [11] and weaned piglets [12]. Numerous studies have demonstrated that the diarrhea incidence of piglets was significantly increased in the LT environment [13]. The present study showed that LT increased the diarrhea incidence and diarrhea index of weaned piglets during the first week, but there were no significant effects on diarrhea of pigs during the second and third week. This indicates that weaned piglets may gradually adapt to cold exposure.

Nutrient digestibility is an important indicator for assessing the digestive capacity of animal gastrointestinal tracts. Studies have shown that LT reduces the proximal gastric diastolic function and accelerates the gastric emptying of animals, resulting in lower digestibility [14]. In the present study, LT decreased the apparent digestibility of CP, OM and DM. Thus, our data supports that cold environments would increase the diarrhea incidence of weaned piglets, leading to dysfunction of the digestive tract, which is also one of the key factors that reduces the apparent digestibility of nutrients.

The mammalian skeletal muscle can be divided into four different *MyHC* isoforms [5]. Several studies have shown that the total number of pig muscle fibers after birth basically does not change, rather, the composition of muscle fiber type changes [15,16]. The types of muscle fiber can be transformed from fast-twitch to slow-switch or slow-switch to fast-twitch by a variety of factors, including innervation, exercise, hormones and ambient temperature [17,18]. The greater abundance of fast oxidative-glycolytic *MyHC IIa* and *MyHC IIx* fibers in the psoas muscle was associated with superior meat quality traits, and the longissimus dorsi muscle are mainly composed of fast glycolytic *MyHC IIb* fibers, which could account for less favourable quality traits [19]. Our study aimed at evaluating the bare effects of cold exposure on muscle fiber types of the oxidative PM and glycolytic LM. In cold environments, pigs mainly produce heat through skeletal muscle contraction, and then maintain their body temperature [20,21]. It has been reported that skeletal muscle contractile activity might lead to muscle fiber type transformation [22]. Previous study had shown that the proportion of oxidative fibers is greater in skeletal muscle from piglets in a cold environment compared with a warm environment, under conditions of controlled food intake [23]. Bee et al. demonstrated that the LM of outdoor pigs during the winter (5 °C) had more fast oxidative-glycolytic fibers and fewer fast glycolytic fibers than muscles of indoor-housed pigs (22 °C) [24]. Similar to previous research results, we found that the proportion of *MyHC IIa* mRNA levels in LM was increased in the LT group. Moreover, the finishing pigs and early postnatal pigs in cold environments have a greater proportion of oxidative fibers in semispinalis muscle (oxidative), compared with pigs at room temperature [12,25]. Gentry et al. demonstrated that the LM muscle of pigs born outdoors (5 °C) had a higher percentage of type I than pigs born indoors (18 °C), and pigs reared outdoors had a lower percentage of IIX fibers than pigs reared indoors for the semispinalis muscle [26]. In this study, the proportion of *MyHC I* mRNA levels was increased in 15 °C compared with that under 26 °C. Therefore, our results support that low-temperature may promote muscle fiber type transformation from type II to type I due to the skeletal muscle continuous contraction caused by high frequency chills.

Mitochondria is the organelle where the main nutrients finally release energy by oxidation, which plays an important role in maintaining the balance of various physiological activities in cells [27]. CS is one of the key enzymes in the production and metabolic pathway of energy, and it has been widely used to evaluate metabolism landmarks of oxidative and respiratory capacity [28]. CPT-1b is located on the outer membrane of mitochondria, which is a key rate-limiting enzyme in fatty acid oxidation in mitochondria [29]. SDHB can be used as a marker enzyme reflecting mitochondrial function. Previous studies have found that cold exposure increased the activities of CS in early postnatal pig muscle longissimus lumborum (LL) and rhomboideus (RH) muscle [25], and SDH activity in skeletal muscle was increased in rats exposed to cold [30]. Similar results were observed in this study that LT increased the mRNA expression of CS in LM and PM. We also found that LT increased the mRNA expression of SDHB in LM muscle and CPT-1b in PM muscle. LM and PM mitochondria exhibit specific changes that are probably involved in the difference of skeletal muscle oxidative metabolism. A previous study showed that muscle fiber types are closely related to muscle mitochondrial synthesis and function [31]. Compared with type II fibers, type I fibers have higher mitochondrial content and oxidative metabolism capacity [6]. These results suggested that cold environment regulated muscle fiber types, which may occur through mitochondrial function regulation.

The antioxidant system is a complete system for scavenging free-radicals that could cause damage to membranes and tissues [32]. T-AOC is an important indicator reflecting the coordination of antioxidant systems in the body. T-SOD is the important antioxidant enzyme and has strong free-radical scavenging ability [33]. Studies have found that SOD activity was decreased in the blood of broilers exposed to the cold environment at 4 °C [34]. The antioxidant enzyme activity and free-radical scavenging ability declined when animals were under cold stress (10 days of 4 °C) [35]. Furthermore, it has been reported that chronic stress induces an increase in T-AOC activity, and the chicken’s T-AOC and T-SOD decreased during acute stress [36]. We found that LT increased the activities of T-AOC in serum and LM, and increased the activities of T-SOD in PM of weaned piglets. LM, PM and serum antioxidative capacity changes are probably involved in the differences between samples characteristics. However, the result was different from previous study, presumably caused by higher temperature or chronic stress. This result indicates that chronic cold stress at 15 °C could elevate antioxidative capacity in weaning piglets. However, the antioxidative capacity would decrease when the temperature is lower than a certain threshold.

## 5. Conclusions

In summary, low-temperature could negatively affect the piglet growth performance and nutrient digestibility. Moreover, we also provided evidence that low-temperature induces a shift toward oxidative muscle fibers, which may occur through mitochondrial function regulation and increased antioxidative capacity.

## Figures and Tables

**Table 1:** Composition and calculated nutrient content of the basal diet.

Ingredients	Content (%)
Extruded corn	30.00
Corn	25.00
Soybean meal	10.50
Extruded soybean	5.50
Rice screenings	8.15
Wheat bran	1.50
Soybean protein concentrate	4.00
Spray-dried animal plasma	1.50
Fish meal	3.50
Whey powder (low protein)	3.80
Soybean oil	2.00
Sucrose	2.00
Limestone	0.88
Dicalcium phosphate	0.40
NaCl	0.30
L-Lysine HCl (78%)	0.42
DL-Methionine	0.14
L-Threonine (98.5%)	0.06
Chloride choline	0.10
Vitamin premix ^a^	0.05
Mineral premix ^b^	0.20
Nutrition level	
DE (Mcal/kg)	3.50
Crude protein	18.03
Calcium	0.80
Total phosphorus	0.56
Available phosphorus	0.36
Lysine	1.35
Methionine	0.44
Threonine	0.79
Tryptophan	0.24

^a^ The premix provided the following per kg of diets: 8000 IU of V_A_, 2000 IU of VD_3_, 20 IU of V_E_, 2 mg of VK_3_, 1.50 mg of VB_1_, 5.6 mg of VB_2_, 1.5 mg of VB_6_, 0.02 mg of VB_12_, 15 mg of niacin, 10 mg of pantothenic acid, 0.60 mg of folic acid and 0.10 mg of biotin. ^b^ The premix provided the following per kg of diets: 100 mg of Fe, 100 mg of Cu, 100 mg of Zn, 20 mg of Mn, 0.30 mg of I and 0.30 mg of Se.

**Table 2:** The primer sequences used for real-time quantitative PCR.

Gene Name	Primer	Sequences (5′→3′)	Product Length (bp)	Accession No.
*β-actin*	Forward	GCGGCATCCACGAAACTAC	138	DQ-845171.1
	Reverse	TGATCTCCTTCTGCATCCTGTC		
*MyHC I*	Forward	AAGGGCTTGAACGAGGAGTAGA	114	AB-053226
	Reverse	TTATTCTGCTTCCTCCAAAGGG		
*MyHC IIa*	Forward	GCTGAGCGAGCTGAAATCC	137	AB-025260
	Reverse	ACTGAGACACCAGAGCTTCT		
*MyHC IIx*	Forward	AGAAGATCAACTGAGTGAACT	149	AB-025262
	Reverse	AGAGCTGAGAAACTAACGTG		
*MyHC IIb*	Forward	ATGAAGAGGAACCACATTA	166	AB-025261
	Reverse	TTATTGCCTCAGTAGCTTG		
*CS*	Forward	GGAAGTCGGCAAAGATGTGT	162	NM-214276.1
	Reverse	TCATGAGGCAGGTGTTTCAG		
*CPT-1b*	Forward	GCTATCTGTGTCCGCCTTCT	151	NM-001007191.1
	Reverse	GGCTGTATTCCTCGTCATCC		
*SDHB*	Forward	TGTGGTCCTATGGTGTTGGA	168	NM-001104953.1
	Reverse	TTTGTCGAGGTTGGTGTCAA		
*Nrf-1*	Forward	TCCATCAATCCGGAAGAGAC	170	XM-021078993.1
	Reverse	GCACCACATTCTCCAAAGGT		

**Table 3:** Effects of low-temperature on growth performance in weaned piglets.

Item	Treatments		*p*-Value
	CON ^a^	LT ^b^	
ADG, g	269.21 ± 20.93	323.60 ± 36.41	0.214
ADFI, g	428.49 ± 24.95	557.72 ± 48.66	0.036
F/G	1.61 ± 0.05	1.77 ± 0.07	0.073

^a^ CON, thermoneutral temperature; ^b^ LT, low-temperature.

**Table 4:** Effects of low temperature on diarrhea in weaned piglets.

Item	Treatments		*p*-Value
	CON	LT	
1–7 d			
Diarrhea incidence (%)	31.42 ± 3.40	52.38 ± 3.99	0.002
Diarrhea index	0.47 ± 0.07	0.90 ± 0.09	0.003
8–14 d			
Diarrhea incidence (%)	22.86 ± 1.84	23.81 ± 1.59	0.702
Diarrhea index	0.27 ± 0.04	0.33 ± 0.03	0.276
15–21 d			
Diarrhea incidence (%)	17.14 ± 3.60	20.63 ± 3.78	0.516
Diarrhea index	0.23 ± 0.04	0.24 ± 0.04	0.880
1–21 d			
Diarrhea incidence (%)	23.81 ± 2.12	32.28 ± 3.67	0.054
Diarrhea index	0.32 ± 0.04	0.49 ± 0.07	0.051

**Table 5:** Effects of low-temperature on growth performance in weaned piglets.

Item	Treatments		*p*-Value
	CON	LT	
CP ^a^, %	86.13 ± 0.67	83.35 ± 0.73	0.014
DM ^b^, %	90.28 ± 0.24	89.16 ± 0.30	0.012
OM ^c^, %	91.98 ± 0.23	90.96 ± 0.27	0.013
GE ^d^, %	90.60 ± 0.28	89.68 ± 0.33	0.051

^a^ CP, crude protein. ^b^ DM, dry matter. ^c^ OM, organic matter. ^d^ GE, gross energy.

**Table 6:** Effects of low-temperature on antioxidant capacity in serum and skeletal muscles.

Item	Treatments		*p*-Value
	CON	LT	
Serum			
MDA, nmol/mL	3.86 ± 0.18	3.91 ± 0.11	0.805
T-AOC, U/mL	0.87 ± 0.05	1.25 ± 0.09	0.002
T-SOD, U/mL	145.25 ± 4.16	146.50 ± 3.56	0.825
LM			
MDA, nmol/mg prot	0.16 ± 0.01	0.14 ± 0.02	0.402
T-AOC, U/mg prot	0.16 ± 0.02	0.27 ± 0.03	0.006
T-SOD, U/mg prot	39.31 ± 1.06	42.93 ± 1.88	0.121
PM			
MDA, nmol/mg prot	0.24 ± 0.02	0.26 ± 0.02	0.466
T-AOC, U/mg prot	0.20 ± 0.02	0.24 ± 0.03	0.340
T-SOD, U/mg prot	35.11 ± 1.53	40.19 ± 1.62	0.039

## Data Availability

The data are available on request from the corresponding author.

## References

[1] Mendes A.A., Watkins S.E., England J.A., Saleh E.A., Waldroup A.L., Waldroup P.W. (1997). Influence of dietary lysine levels and arginine:lysine ratios on performance of broilers exposed to heat or cold stress during the period of three to six weeks of age. Poult. Sci..

[2] Li J., Huang F., Li X., Su Y., Li H., Bao J. (2017). Effects of intermittent cold stimulation on antioxidant capacity and mRNA expression in broilers. Livest. Sci..

[3] Janský L. (2010). Non-shivering thermogenesis and its thermoregulatory significance. Biol. Rev..

[4] Henneman E., Clamann H.P., Gillies J.D., Skinner R.D. (1974). Rank order of motoneurons within a pool: Law of combination. J. Neurophysiol..

[5] Lefaucheur L., Hoffman R.K., Gerrard D.E., Okamura C.S., Rubinstein N., Kelly A. (1998). Evidence for three adult fast myosin heavy chain isoforms in type II skeletal muscle fibers in pigs. J. Anim. Sci..

[6] Berchtold M.W., Brinkmeier H., Muntener M. (2000). Calcium ion in skeletal muscle: Its crucial role for muscle function, plasticity, and disease. Phys. Rev..

[7] Dividich J.L., Vermorel M., Noblet J., Bouvier J.C., Aumaitre A. (1980). Effects of environmental temperature on heat production, energy retention, protein and fat gain in early weaned piglets. Br. J. Nutr..

[8] McCarthy J., Aherne F., Okai D. (1974). Use of HCl insoluble ash as an index material for determining apparent digestibility with pigs. Can. J. Anim. Sci..

[9] AOAC International (1995). Official Methods of Analysis of AOAC International.

[10] Schmittgen T.D. (2001). Real-time quantitative PCR. Methods.

[11] Stahly T., Cromwell G., Aviotti M. (1979). The effect of environmental temperature and dietary lysine source and level on the performance and carcass characteristics of growing swine. J. Anim. Sci..

[12] Lefaucheur L., Le D.J., Mourot J., Monin G., Ecolan P., Krauss D. (1991). Influence of environmental temperature on growth, muscle and adipose tissue metabolism, and meat quality in swine. J. Anim. Sci..

[13] Kelley K.W., Blecha F., Regnier J.A. (1982). Cold exposure and absorption of colostral immunoglobulins by neonatal pigs. J. Anim. Sci..

[14] Sun S., Huang X., Hou X., Xie X. (2005). Impaired accommodation of the proximal stomach to a colder meal in healthy adults. J. Clin. Intern. Med..

[15] Wigmore P.M., Stickland N.C. (1983). Muscle development in large and small pig fetuses. J. Anat..

[16] Stickland N.C., Handel S.E. (1986). The numbers and types of muscle fibres in large and small breeds of pigs. J. Anat..

[17] Picard B., Lefaucheur L.C., Duclos M.J. (2002). Muscle fibre ontogenesis in farm animal species. Reprod. Nutr. Dev..

[18] Chang K.C. (2007). Key signalling factors and pathways in the molecular determination of skeletal muscle phenotype. Animal.

[19] Chang K.C., Costa N.D., Blackley R., Southwood O., Evans G., Plastow G., Wood J.D., Richardson R.I. (2003). Relationships of myosin heavy chain fibre types to meat quality traits in traditional and modern pigs. Meat Sci..

[20] Heath M., Ingram D.L. (1983). Thermoregulatory heat production in cold-reared and warm-reared pigs. Am. J. Physiol..

[21] Dauncey M.J., Ingram D.L. (1986). Acclimatization to warm or cold temperatures and the role of food intake. J. Therm. Biol..

[22] Bottinelli R., Canepari M., Reggiani C., Stienen G.J. (1994). Myofibrillar ATPase activity during isometric contraction and isomyosin composition in rat single skinned muscle fibres. J. Physiol..

[23] Dauncey M.J., Ingham D.L. (1990). Respiratory enzymes in muscle: Interaction between environmental temperature, nutrition and growth. J. Therm. Biol..

[24] Bee G., Guex G., Herzog W. (2004). Free-range rearing of pigs during the winter: Adaptations in muscle fiber characteristics and effects on adipose tissue composition and meat quality traits. J. Anim. Sci..

[25] Lefaucheur L., Ecolan P., Lossec G., Gabillard J.C., Butler-Browne G.S., Herpin P. (2001). Influence of early postnatal cold exposure on myofiber maturation in pig skeletal muscle. J. Muscle Res. Cell. Motil..

[26] Gentry J.G., Mcglone J.J., Miller M.F., Blanton J. (2004). Environmental effects on pig performance, meat quality, and muscle characteristics. J. Anim. Sci..

[27] Sherratt H.S.A. (1991). Mitochondria: Structure and function. Rev. Neurol..

[28] Spina R.J., Chi M.M., Hopkins M.G., Nemeth P.M., Lowry O.H., Holloszy J.O. (1996). Mitochondrial enzymes increase in muscle in response to 7-10 days of cycle exercise. J. Appl. Physiol..

[29] Bruce C.R., Hoy A.J., Turner N., Watt M.J., Allen T.L., Carpenter K., Cooney G.J., Febbraio M.A., Kraegen E.W. (2009). Overexpression of carnitine palmitoyltransferase-1 in skeletal muscle is sufficient to enhance fatty acid oxidation and improve high-fat diet–induced insulin resistance. Diabetes.

[30] Hannon J.P. (1960). Effect of prolonged cold exposure on components of the electron transport system. Am. J. Physiol..

[31] Herpin S.P. (1997). Postnatal Changes in Mitochondrial Protein Mass and Respiration in Skeletal Muscle from the Newborn Pig. Biochem. Mol. Biol..

[32] Brand-Williams W.M., Cuvelier M.E., Berset C. (1995). Use of free radical method to evaluate antioxidant activity. LWT Food Sci. Technol..

[33] Djordjevi V.B. (2004). Free radicals in cell biology. Int. Rev. Cytol..

[34] Ramnath V., Rekha P. (2009). Brahma Rasayana enhances in vivo antioxidant status in cold-stressed chickens (Gallus gallus domesticus). Indian J. Pharmacol..

[35] Venditti P., Pamplona R., Ayala V., De Rosa R., Caldarone G., Di Meo S. (2006). Differential effects of experimental and cold-induced hyperthyroidism on factors inducing rat liver oxidative damage. J. Exp. Biol..

[36] Zhao F.-Q., Zhang Z.-W., Qu J.-P., Yao H.-D., Li M., Xu S.-W. (2014). Cold stress induces antioxidants and Hsps in chicken immune organs. Cell Stress Chaperones.

